# Vinflunine – an active chemotherapy for treatment of advanced non-small-cell lung cancer previously treated with a platinum-based regimen: results of a phase II study

**DOI:** 10.1038/sj.bjc.6603106

**Published:** 2006-04-25

**Authors:** J Bennouna, J-L Breton, J-M Tourani, C Ottensmeier, M O'Brien, P Kosmidis, T E Huat, M-C Pinel, C Colin, J-Y Douillard

**Affiliations:** 1 Centre René Gauducheau, Boulevard Jacques Monod, Saint-Herblain 44805, France; 2 Centre Hospitalier Général, Belfort, France; 3 Centre Hospitalier Universitaire, Poitiers, France; 4 Southampton University Hospitals, Southampton, UK; 5 Royal Marsden Hospital, Sutton, UK; 6 Hygeia Hospital, Athens, Greece; 7 National Cancer Center, Singapore, Singapore; 8 Institut de Recherche Pierre Fabre, Boulogne-Billancourt 92654, France

**Keywords:** vinflunine, phase II study, non-small-cell-lung-cancer

## Abstract

A multicentre, single-arm, phase II trial designed to determine the efficacy of single-agent vinflunine in patients with advanced non-small-cell lung cancer (NSCLC) previously treated with a platinum-based regimen. The objectives were to assess efficacy in terms of tumour response rate (primary end point), duration of response, progression-free survival (PFS) and overall survival (OS), and to evaluate the toxicity associated with this treatment. Patients with advanced NSCLC with progressive disease having failed prior platinum-based first-line treatment for advanced disease. Five responses out of the 63 treated patients were documented by WHO criteria and validated by an independent panel review (IRP), yielding a response rate of 7.9% (95% CI: 2.6–17.6) in the intent-to-treat analysis and 8.3% (95% CI: 2.8–18.4) in the evaluable population. Disease control was achieved in 35 out of 60 evaluable patients (58.3%). The median duration of response (complete response+partial response), according to modified WHO criteria was 7.8 months (95% CI: 4.6–NR). Median PFS was 2.6 months (95% CI: 1.4–3.8), and the median survival was 7.0 months (95% CI: 5.8–9.2). Grades 3–4 neutropenia was reported in 50% of patients; febrile neutropenia was observed in two patients (3.2%); grades 3–4 myalgia and grade 3 constipation were experienced by 10 (15.9%) and six (9.5%) of patients, respectively. Constipation was manageable, noncumulative and could be prevented with laxative prophylaxis. The encouraging results from this phase II study with vinflunine warrant further investigations in phase III trials as second- or first-line treatment of advanced non-small-cell lung carcinoma, as a single agent or in combination with other active drugs.

Patients with newly diagnosed inoperable stage (IIIB–IV) lung cancer and reasonable performance status should receive treatment with at least one chemotherapy regimen and it is now accepted that the schedule should be platinum based, in combination with a newer agent such as a taxane, vinorelbine or gemcitabine (Kelly *et al*, 2001; Schiller *et al*, 2002). The benefits of this approach are relatively modest; however, patients who progress on or after first-line chemotherapy for advanced disease but continue to be in good general condition may be offered second-line chemotherapy. Currently, three drugs are approved for second-line therapy of non-small-cell lung cancer (NSCLC) (docetaxel, erlotinib and pemetrexed), however, the life expectancy of these patients remains short and new drugs are urgently needed for this setting.

Vinflunine (Javlor®, Pierre Fabre Médicament, Boulogne-Billancourt, France) is a novel tubulin-targeted agent obtained by semisynthesis using superacidic chemistry to selectively introduce two fluorine atoms at the 20′ position of catharanthine moiety of vinca alkaloid (Fahy *et al*, 1997). The actions of vinflunine on microtubules produce effects on mitotic spindle functions leading to modifications of cell cycle progression and cell killing (Ngan *et al*, 2000) Vinflunine prevents microtubule assembly during mitosis (Etiévant *et al*, 1998; Kruczynski *et al*, 1998; Ngan *et al*, 2001). The affinity profile of vinflunine shows features which suggest that it will have greater effects on mitotic rather than axonal tubulin and so will not cause significant neurotoxicity (Lobert *et al*, 1998). Vinflunine showed antitumour activity in xenografts tested and high *in vivo* activity against the NCI H69 human NSCLC model (Hill *et al*, 1999; Kruczynski *et al*, 1999). Vinflunine treatment every 3 weeks was considered optimal, based on clinical and pharmacokinetic data from the three dose schedules evaluated in Phase I trials. Vinflunine was administered at the beginning of the phase I trials at 350 mg m^−2^ in normal saline as a 10-min infusion; after an early safety analysis this dose was adjusted at 320 mg m^−2^ which is the recommended dose for all subsequent patients included in clinical trials (Bennouna *et al*, 2003). The preclinical profile of vinflunine suggests that this first fluorinated antitubulin compound is a good candidate for second-line chemotherapy in patients with advanced NSCLC cancer after failure of standard platinum-based chemotherapy.

## MATERIALS AND METHODS

### Objectives

This study was an open-label, multicentre, nonrandomised phase II trial designed to determine the efficacy of vinflunine in patients with advanced NSCLC with clear evidence of metastatic disease who had failed platinum-based therapies for advanced disease. The primary objective was to measure response rate by WHO criteria; secondary objectives were to assess duration of response, progression-free survival (PFS) and overall survival (OS) and to evaluate the toxicity associated with this treatment. The protocol and its amendments were submitted to Independent Ethics Committees (IEC) according to local requirements. The study was conducted in accordance with the ethical principles set forth in the Declaration of Helsinki (Somerset West 1996) and in compliance with Good Clinical and Laboratory Practices. Written informed consent was obtained from each participating patient prior to entry into the study.

### Patient selection

Patients were recruited from seven active centres between October 2001 and May 2003. Patients eligible for the study were required to have a histologically confirmed diagnosis of NSCLC cancer with clear radiological or clinical evidence of progressive disease (PD). Previous systemic chemotherapy or radiotherapy had to have been stopped 30 days before the administration of the study drug, and a full recovery from all side effects was necessary. The presence of at least one bidimensionally measurable lesion, not previously irradiated, assessed by CT-scan or MRI performed <28 days before 1st day of study drug administration was required. Patients were required to be aged ⩾18 years with Karnofsky Performance Status (KPS)⩾80 and an estimated life expectancy of ⩾12 weeks. Evidence of adequate haematological function (absolute neutrophil count (ANC) ⩾2.0 × 10^9^ l^−1^, platelets ⩾100 × 10^9^ l^−1^), hepatic function (bilirubin ⩽1.5 × upper normal limit (UNL), transaminases ⩽2.5 × UNL, unless due to liver involvement), normal renal function and a normal ECG was required.

### Treatment schedule

Vinflunine was given at the dose of 320 mg m^−2^ as a 10-min infusion every 21 days. Tolerance was assessed throughout the treatment period and before each administration according to the NCI Common Toxicity Criteria (Version 2.0). All patients who received at least one vinflunine administration were considered as evaluable for safety. The use of haematopoietic growth factors (G-CSF) was allowed for patients with febrile neutropenia or neutropenic infections according to local guidelines. Vinflunine had to be delayed by 1 or 2 weeks in the case of haematological or nonhaematological toxicity (grade >2 toxicity impacting a major organ except for alopecia). If febrile neutropenia and/or a grade 4 neutropenia (< 1.0 × 10^9^ l^−1^) lasting 7 days or more was observed between 2 subsequent administrations of vinflunine, the dose was reduced to 280 mg m^−2^ from the next cycle on. If, after a dose reduction, this toxicity was seen again with the same severity, the dose was further reduced to 250 mg m^−2^. If at this dose level the same event recurred, the treatment was stopped. Blood counts were to be performed every 2 days until recovery of ANC ⩾1.0 × 10^9^ l^−1^. No dose re-escalation was allowed after dose reduction. In case of grade 2 mucositis and/or constipation lasting more than 5 days, or grade ⩾3 mucositis, and/or constipation of any duration, the vinflunine dose was reduced to 280 mg m^−2^ from the next cycle on. If, after dose reduction, one of these toxicities was seen again, the dose was reduced to 250 mg m^−2^. If at this dose the event recurred, the treatment was discontinued. Each patient had to receive at least two cycles of treatment unless early progression, unacceptable toxicity, serious intercurrent illness, other reactions occurred which could affect the clinical status of the patient to a significant degree requiring discontinuation of the drug, or request by the patient to withdraw consent.

After the initial two cycles, tumour response was assessed; for patients showing PD the treatment had to be discontinued; patients showing stable disease (SD) received two further cycles of vinflunine, after a second assessment treatment could be continued at the Investigator's discretion; patients presenting with a complete or a partial response (CR, PR) could continue treatment until PD, toxicity or patient preference precluded further therapy.

### Baseline and treatment evaluation

Preregistration assessments included a detailed medical history, CT-scan, MRI or physical examination (in case of superficial lymph node or skin nodule) for tumour assessment. All positive imaging procedures at study entry had to be repeated every 6 weeks. An assessment of symptoms was made at study entry and then throughout treatment. Physical examination and vital signs were assessed on day one of each cycle. A complete blood cell count (including a differential and platelet count) was taken at baseline (within a maximum of 7 days prior to study drug administration) and during treatment, before each cycle. Additional samplings were planned on day 8s and 15 of each cycle: in case the ANC was < 1.0 × 10^9^ l^−1^, counts were repeated every 2 days until recovery to ANC ⩾1.0 × 10^9^ l^−1^. Transaminases, alkaline phosphatases, total bilirubin, lactate dehydrogenase, creatinine, electrolytes including Ca^2+^, Na^+^ and K^+^ and total protein were assessed at every cycle. Electrocardiogram was to be performed and recorded prior to initial administration and repeated at every cycle.

If PD had not occurred during the study treatment period, all lesions were regularly assessed until disease progression. Efficacy was assessed by investigators and subsequently by an Independent Review Panel (IRP) using both the WHO criteria (World Health Organisation, 1979; Green and Weiss, 1992) and RECIST guidelines (Therasse *et al*, 2000); CR was defined as disappearance of all lesions clinically and radiologically. Partial response was defined as ⩾50% reduction in the sum of the products of perpendicular diameters of all lesion measurements maintained for at least 4 weeks while no other type of lesion progressed or appeared. Stable disease was defined as <50% reduction and <25% increase in the sum of the products of perpendicular diameters of all lesion measurements maintained for at least 4 weeks while no other type of lesion progressed or appeared. All responses and disease stabilisations (if appropriate) were reviewed by an independent radiologist. The duration of response was calculated for patients with confirmed response (CR or PR) from the date of registration until the date of documented progression, start of new anticancer therapy, date of death, lost follow-up or last news. The PFS was defined as the time elapsed from registration until progression, death, lost follow-up or last news; survival was defined as the time elapsed from registration date to death or lost to follow-up, or last date the patient was known to be alive.

### Statistical analysis

Sample size was based on a one-sample multiple testing procedure (Fleming, 1982). With 55 evaluable patients, a null hypothesis for the true response rate of 4% and an alternative hypothesis of 14%, the type I error *α* was 5% and the type II error *β* was less than 20%.

Continuous data were summarised using median, minimum and maximum values. Categorical data were presented in contingency tables with frequencies and percentages. Exact confidence intervals were calculated at the 95% level. Time dependent parameters were analysed using the Kaplan–Meier method and 95% confidence interval for the median was reported.

Efficacy analyses were performed on the intent to treat and evaluable population. The primary efficacy parameter was response rate and included only confirmed CR and PR. The other efficacy parameters were duration of response, PFS and OS.

Safety analyses were performed on the population of patients having received at least one dose of study treatment. Worst NCI CTC grade for haematological and nonhaematological adverse events were presented.

All statistical analyses were carried out with 8.2 version of SAS® (SAS Institute Inc., Cary, NC, USA) for Windows®.

## RESULTS

Sixty-six patients with advanced or metastatic NSCLC were included in this study. Three patients who were included but not treated as they presented with intercurrent serious conditions after signing informed consent, were not included in the analysis. In line with the ICH E9 guidelines in which it states that only treated patients should be reported, the intent-to-treat analysis therefore includes 63 patients; an additional three patients were found to be ineligible after enrolment, hence results will also be presented for the 60 evaluable patients.

### Evaluation of efficacy

Demographic features of the patients are summarised in Table 1. As planned, all patients had previously received chemotherapy including platinum (cisplatin or carboplatin or both). All but two patients had received this chemotherapy for advanced disease. The median treatment-free interval after platinum-based chemotherapy was 4.4 months (range 0.5–30.2 months). All patients enrolled in the study had clear evidence of PD, 74.6% had two or more metastatic lesions at entry. According to WHO criteria, five responses out of the 63 treated patients were documented and validated by an IRP, yielding a response rate of 7.9% (95% CI: 2.6–17.6) in the intent-to-treat analysis and 8.3% (95% CI: 2.8–18.4) in the evaluable population. The previous treatment of these patients consisted of carboplatin plus paclitaxel for four patients and cisplatin plus gemcitabine for one patient. The response rate was also analysed by the IRP using RECIST criteria: six patients were assessed as responders, yielding a response rate of 9.5% (95% CI: 3.6–19.6) in the intent-to-treat analysis and 10% (95% CI: 3.8–20.5) on the evaluable population as shown in Table 2. Disease control, that is the absence of PD (CR+PR+SD by WHO criteria) was achieved in 35 patients out of the 60 evaluable (58.3%) patients.

**Figure 1:**
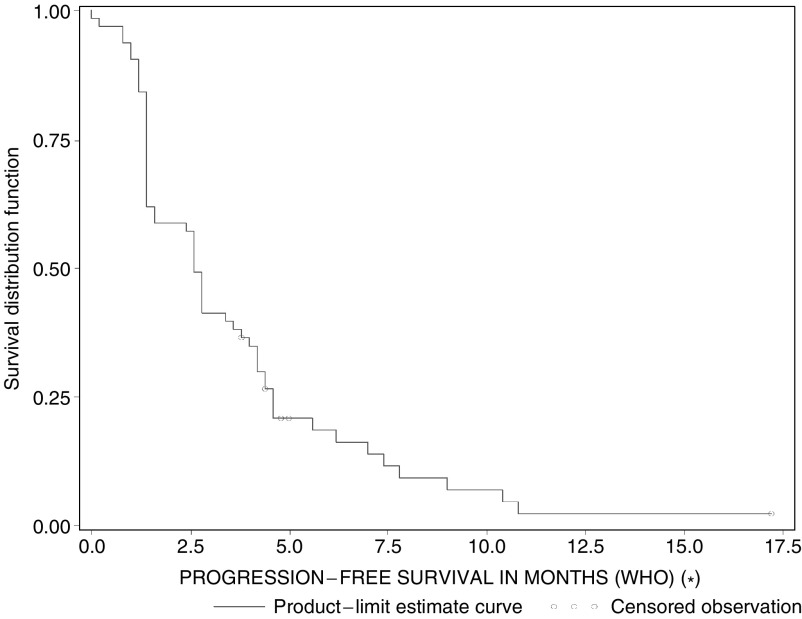
Progression-free survival.

**Figure 2:**
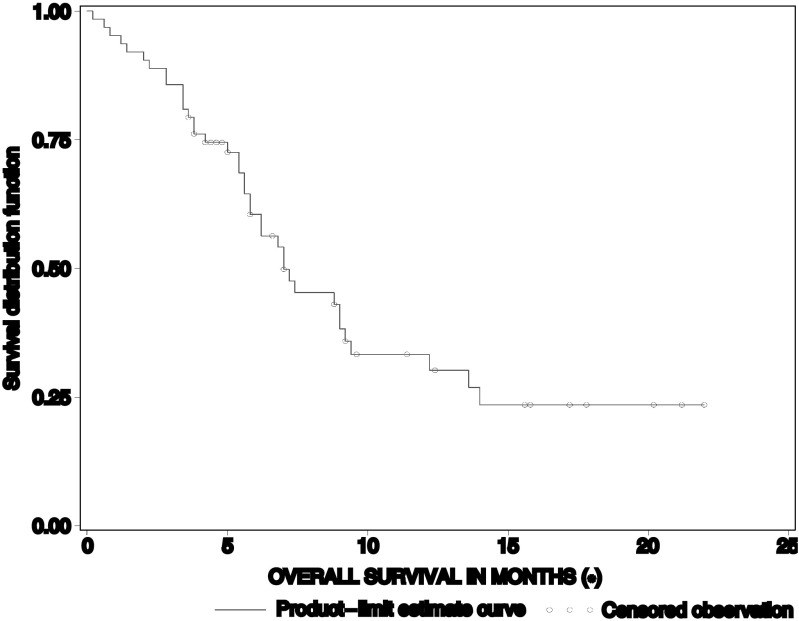
Overall survival.

The median duration of response (CR+PR), according to WHO criteria was 7.8 months (95% CI: 4.6–NR). Median PFS was 2.6 months (95% CI: 1.4–3.8). The median survival time was 7 months (95% CI: 5.8–9.2) (Figures 1 and 2). Twenty-two patients (34.9%) received at least one further chemotherapy after study discontinuation (16 patients received monotherapy as follows: docetaxel: 12 patients; gemcitabine: two patients; irinotecan: one patient; and experimental agent: one patient; six patients underwent further chemotherapy with a combination regimen; cisplatin–vinorelbine: two patients; gemcitabine–docetaxel, gemcitabine–carboplatin, mitomycin C–vinblastine–cisplatin and docetaxel–carboplatin: one patient, respectively).

Karnofsky Performance Status was recorded for each patient at baseline and before each treatment cycle during the study (i.e. every 3 weeks). Ten out of 63 patients (15.9%) had an improvement of their KPS during treatment and 36 (57.1%) maintained their baseline KPS. Only 15 patients (23.8%) had a worsening KPS during treatment.

### Evaluation of safety

The total number of cycles delivered was 223 (median number was 2 (range 1–9)). The median relative dose intensity by patient was 99.4% of the theoretical scheduled dose. Seventeen out of 63 patients (27%) (32.5% of cycles) had dose reductions. Fourteen patients (22.2%) had at least one cycle delayed; 20 cycles were postponed as follows: five delays ⩾4 days and <7 days, 11 delays between 7 and 14 days and four delays ⩾14 days. Six delays were due either to drug related haematological toxicity (four) or to drug-related nonhaematological toxicity (two); the other 14 cycles were postponed due to adverse events unrelated to the study drug and logistical or administrative issues.

Grade 3/4 neutropenia was observed in 50% of patients but only two patients experienced febrile neutropenia (3.2%), and 3.2% neutropenic infection. No prophylactic growth factors were allowed. Although growth factors were permitted for the treatment of febrile neutropenia or grade 4 neutropenia without infection no patient in the study received this intervention. grade 3 anaemia was observed in 6.5% of patients but no grade 4 anaemia was reported; thrombocytopenia was rare (Table 3). Grade 3/4 myalgia was observed in 15.9% of patients; six out 63 patients suffered from grade 3 constipation (9.5%) that was reported in five patients after the first dose; no grade 4 constipation was reported. Severe fatigue, arthralgia anorexia, abdominal pain and stomatitis were uncommon and observed in <6% of patients (Table 4).

No clinical significant alterations in biological parameters were observed. (creatinine, total bilirubin, SGOT/AST, SGPT/ALT and alkaline phosphatase).

## DISCUSSION

Although in 1997, ASCO guidelines stated that there was no current evidence that second-line chemotherapy improved survival with advanced NSCLC (American Society of Clinical Onology, 1997) there are now a number of trials which have demonstrated benefit for patients with disease progression after first-line chemotherapy (Biesma *et al*, 1999) including a number of studies which have documented a quality of life benefit (Dancey *et al*, 1999; Miller *et al*, 1999).

Results of the phase II study presented here have demonstrated that vinflunine has clinically relevant activity as second-line treatment of patients with NSCLC who have failed first-line treatment with a platinum-containing regimen. The overall response rate of 8% and a median OS of over 7 months compares favourably with reports in the literature on the use of best supportive care and of other new agents. At the current time only erlotinib, docetaxel and pemetrexed are approved for the treatment of advanced, platinum-refractory non-small lung cancer. The application for broadening the indication of docetaxel to include second-line chemotherapy of patients with locally advanced or metastatic NSCLC was based on the results of a phase III study of docetaxel *vs* vinorelbine/ifosfamide (Fossella *et al*, 2000) and another phase III study of docetaxel *vs* supportive care. The first study (Shepherd *et al*, 2000) failed to show a significant effect on the primary end point (OS), while it showed a significant increase in one secondary end point, the response rate, which was 10.5 and 6.5% for docetaxel 100 and 75 mg m^−2^, respectively, compared with 0.8% in the vinorelbine/ifosfamide. The study comparing docetaxel with supportive care in previously treated patients was analysed in two parts corresponding to two successive periods and doses of docetaxel: 100 and 75 mg m^−2^. In this study a significant increase in OS (*P*=0.016) was observed. Docetaxel treatment also showed positive effects in several secondary endpoints of this study including the overall response rate of 6%; time to progression was significantly improved in the overall docetaxel group (10.6 *vs* 6.7 weeks), as well as in docetaxel 75 mg m^−2^ (12.3 *vs* 7 weeks) and in docetaxel 100 mg m^−2^ subgroups (9.1 *vs* 5.9 weeks). The lower dose was generally better tolerated than in the higher dose. Recently, pemetrexed has demonstrated an overall response rate of 9% and 8-month median survival in a phase III trial comparing this drug with docetaxel; differences with the latter were generally limited to toxicity, with pemetrexed-treated patients experiencing less myelosuppression and fewer hospitalisations (Hanna *et al*, 2004). The double-blind placebo-controlled trial of erlotinib has shown that this drug significantly increases the PFS by 2 weeks and the OS by 2 months in comparison with best supportive care and placebo; the median OS of 6.7 months in erlotinib-treated patients in this trial is comparable with the results of the other agents (Shepherd *et al*, 2005). The role of gemcitabine and ixabepilone in the management of NSCLC patients who have failed first-line therapy is not yet clear due to the lack of phase III trial data in this setting (Rosvold *et al*, 1998; Vansteenkiste *et al*, 2003).

The toxicity profile of vinflunine is also acceptable by comparison with the other candidate agents; the most frequent adverse events observed were neutropenia in 50% of patients; however, only 3.2% developed febrile neutropenia; grade 3 constipation was observed in 9.5% of patients, but was manageable, noncumulative and could be prevented with prophylactic treatment.

The encouraging results from this phase II study with vinflunine warrant further investigations in phase III trials as second or first line treatment of advanced non-small cell lung carcinoma, as a single agent or in combination with other active drugs. Currently, a phase III trial comparing vinflunine with docetaxel in second-line treatment of NSCLC is ongoing.

## Figures and Tables

**Table 1:** Demographic data

*Age (years)*		
Median	61.6	
Range	(38.7–78.7)	
	Patients (n=63)	%
*Sex*		
Male	47	74.6
Female	16	25.4

*Karnofsky PS Status*		
100	14	22.2
90	21	33.3
80	28	44.4

*Histopathologic type*		
Adenocarcinoma	35	55.6
Squamous cell carcinoma	18	28.6
Large cell carcinoma	3	4.8
Not specified	7	11.1

*Treatment type*		
Chemotherapy alone	38	60.3
Radiotherapy+chemotherapy	11	17.5
Surgery+chemotherapy	10	15.8
Surgery+radiotherapy+chemotherapy	4	6.4

*Chemotherapy*		
Carboplatin–paclitaxel	15	23.8
Carboplatin–gemcitabine	13	20.6
Carboplatin–docetaxel	4	6.3
Carboplatin–vinorelbine	1	1.6
Cisplatin–vinorelbine	12	19
Cisplatin–gemcitabine	8	12.7
Cisplatin–mitomycin C–vinblastine	6	9.5
Cisplatin–mitomycin C–ifosfamide	2	3.2
Carboplatin–docetaxel–gemcitabine	1	1.6
Cisplatin–vinorelbine–ifosfamide	1	1.6

**Table 2:** Overall response rate

	**ITT population**		**Evaluable population**	
	**WHO**	**RECIST**	**WHO**	**RECIST**
Number of patients	63	63	60	60
Complete response (CR)	—	—	—	—
Partial response (PR)	5 (7.9%)	6 (9.5%)	5 (8.3%)	6 (10%)
Overall response (CR+PR)	5 (7.9%)	6 (9.5%)	5 (8.3%)	6 (10%)
95% Confidence interval	(2.6–17.6)	(3.6–19.6)	(2.8–18.4)	(3.8–20.5)
Stable disease	31 (49.2%)	31 (49.2%)	30 (50%)	30 (50%)
Disease control (CR+PR+NC)	36 (57.1%)	37 (58.7%)	35 (58.3%)	35 (58.3%)
Disease progression (PD)	25 (39.7%)	24 (38.1%)	25 (41.7%)	24 (40%)
Not evaluable	2 (3.2%)	2 (3.2%)		

**Table 3:** Haematological (NTC CTC, Version 2.0) drug-related adverse events

	***N*=62^a^ patients**						***N*=221^a^ cycle**					
	**Overall incidence**		**Grade 3**		**Grade 4**		**Overall incidence**		**Grade 3**		**Grade 4**	
**Haematological**	***n***	**%**	***n***	**%**	***n***	**%**	***n***	**%**	***n***	**%**	***n***	**%**
Anaemia	55	88.7	4	6.5	0	0	177	80.1	4	1.8	0	0
Leucopenia	53	85.5	15	24.2	5	8.1	147	66.5	25	11.3	7	3.2
Neutropenia	51	82.3	16	25.8	15	24.2	150	67.9	35	15.8	23	10.4
Thrombocytopenia	21	33.9	3	4.8	0	0	57	25.8	3	1.4	0	0
Febrile neutropenia	2	3.2	2	3.2	0	0	2	0.9	2	0.9	0	0
Infection with G3/4 neutropenia	2	3.2	2	3.2	0	0	2	0.9	2	0.9	0	0

a One patient was not evaluable for haematological toxicity.

**Table 4:** Non-haematological (NTC CTC,Version 2.0) drug-related adverse events

	***N*=63 pts**						***N*=223 cycle**					
	**Overall incidence**		**Grade 3**		**Grade 4**		**Overall incidence**		**Grade 3**		**Grade 4**	
**Nonhaematological**	***n***	**%**	***n***	**%**	***n***	**%**	***n***	**%**	***n***	**%**	***n***	**%**
Cardiac ischaemia	1	1.6	0	0	1	1.6	1	0.4	0	0	1	0.4
Thrombosis/embolism	1	1.6	0	0	1	1.6	2	0.9	0	0	2	0.9
Fatigue	34	54.0	3	4.8	1	1.6	85	38.1	3	1.3	1	0.4
Anorexia	10	15.9	2	3.2	0	0	12	5.4	2	0.9	0	0
Constipation	40	63.5	6	9.5	0	0	93	41.7	6	2.7	0	0
Gastritis	3	4.8	1	1.6	0	0	5	2.2	1	0.4	0	0
Stomatitis	26	41.3	2	3.2	1	1.6	42	18.8	2	0.9	1	0.4
Abdominal pain	18	28.6	2	3.2	0	0	27	12.1	3	1.3	0	0
Arthralgia	3	4.8	3	4.8	0	0	4	1.8	3	1.3	0	0
Chest pain	8	12.7	1	1.6	0	0	12	5.4	1	0.4	0	0
Myalgia	18	28.6	8	12.7	2	3.2	45	20.2	9	4	2	0.9
Neuropathic pain	9	14.3	1	1.6	0	0	18	8.1	1	0.4	0	0
2	2	3.2	1	1.6	0	0	3	1.3	1	0.4	0	0
ARDS	1	1.6	0	0	1	1.6	1	0.4	0	0	1	0.4
